# The Value of Hepatic Scintigraphy in the Diagnosis of Biliary Atresia

**DOI:** 10.3389/fped.2022.874809

**Published:** 2022-05-31

**Authors:** Wing Ki Chan, Patrick Ho Yu Chung, Kenneth Kak Yuen Wong

**Affiliations:** ^1^Li Ka Shing Faculty of Medicine, The University of Hong Kong, Hong Kong, China; ^2^Department of Surgery, The University of Hong Kong, Hong Kong, China

**Keywords:** Biliary Atresia (BA), laparoscopy, neonatal cholestasis, Kasai portoenterostomy, hepatic scintigraphy

## Abstract

**Introduction:**

Biliary Atresia (BA) requires prompt diagnosis and surgical intervention to optimize its outcome. The aim of this study was to evaluate the accuracy of EHIDA in distinguishing between BA and other causes of cholestatic jaundice.

**Methods:**

This was a retrospective study of all patients who underwent EHIDA in a tertiary center from 1997 to 2019. The sensitivity, specificity, Negative Predictive Value (NPV) and Positive Predictive Value (PPV) of EHIDA were evaluated. Factors that can potentially affect its accuracy were also analyzed.

**Results:**

During the study period, 93 patients aged 10 to 110 days with cholestasis and suspected BA underwent EHIDA. The sensitivity and NPV were 91.2 and 85.3% while specificity and PPV were 80.6 and 88.1%. These results suggested that EHIDA is suboptimal in both diagnosing or excluding BA. Out of 59 patients who showed no tracer activities in the intestines after 24 h, 56 were subjected to surgical exploration and 52 (92.9%) were eventually diagnosed BA. The accuracy of EHIDA scan were different by the maturity of the patient, age at testing and severity of cholestasis.

**Conclusions:**

EHIDA has a limited accuracy and surgical exploration remains the gold standard to establish the diagnosis of BA. Potential confounding factors that may affect the accuracy of EHIDA were identified but require further studies with larger sample sizes to validate.

## Introduction

Biliary Atresia (BA) is a congenital disorder characterized by destructive inflammatory obliterative cholangiopathy of the entire biliary system resulting in life-threatening cholestasis (1). BA is one of the most challenging liver diseases in infants and a prompt diagnosis is required for the best outcome. There have been controversies regarding the diagnostic pathway for BA. The current gold standard for the diagnosis of BA is surgical exploration with or without liver biopsy to demonstrate the presence of an atretic bile duct as well as peribiliary fibrosis and ductular proliferation. A high sensitivity of 100%, specificity of 94.3% and an accuracy rate of 96.9% have been reported for this diagnostic approach (2, 3). Over the years, there is still no single non-invasive test that can effectively diagnosis BA. Hepatic scintigraphy (EHIDA) is a functional radioisotope study that has commonly been performed as one of the investigations for the evaluation of neonatal cholestasis. Some centers depend on the result of EHIDA scan for the decision to initiate surgical referral for suspected BA. The study required intravenous administration of radioisotopes such as Tc-99m Mebrofenin and Disofenin. The hepatic uptake, excretion of tracer via the liver, bile ducts and intestines are subsequently evaluated. The absence of tracer activity in the intestines after 24 hours is a feature suggestive of extrahepatic occlusive disorders including BA (4). A previous study reported that the sensitivity and specificity of EHIDA was 98.7% and 37–74% respectively, revealing a doubtful reliability (5). Furthermore, misinterpretation of EHIDA scan has also been reported (6). The potential inaccuracy of EHIDA may lead to a delayed Kasai portoenterostomy. It was estimated that about 70% of the BA patients can establish bile flow if KPE is performed within the first 60 days of life; while only 25% of them can restore biliary drainage if KPE is performed after 90 days of life (3). Furthermore, treatment is different for other non-BA causes of neonatal cholestasis and therefore it is important to differentiate between the causes of cholestatic jaundice (7). The aim of this study was to evaluate the diagnostic accuracy of EHIDA in patients with suspected BA due to persistent cholestasis, as well as highlighting the limitations associated with EHIDA scan to reinforce the fact that it should not delay or replace surgical exploration for a diagnosis of BA. Furthermore, we attempted to examine factors that can potentially affect the accuracy of EHIDA scan.

## Methods

This was a retrospective study conducted in a tertiary referral center for BA. All infants aged 10 to 110 days who underwent EHIDA for conjugated hyperbilirubinemia in our institute from September 1997 to October 2019 were included. During the study, each patient was given an intravenous injection of 99m Tc-Mebrofenin. Imaging was then carried out on a Gamma Camera with the patient lying supine. Serial images were acquired at 15 min, 30 min, 45 min and 60 min for 3 h. If there is non-excretion of the radiotracer in the intestines, a delayed image would be acquired at 24 h for confirmation. Non-excretion of 99m Tc-mebrofenin in the intestines in the 24-h delayed image was considered a feature suggestive of BA. This was also the definition of positive EHIDA result in this study. For patients with drainage of radiotracer in the intestines or having inconclusive findings, they were defined as having a negative EHIDA result. In all patients, the diagnosis of BA was confirmed by laparoscopic exploration to reveal the presence of an atretic bile duct. If necessary, an intra-operative cholangiogram was also performed to examine the biliary patency. For the analysis, we determined the sensitivity, specificity, NPV and PPV of EHIDA in diagnosing BA. Factors that can potentially affect the diagnostic accuracy of EHIDA were also analyzed (prematurity, age of life to conduct EHIDA, conjugated bilirubin level latest to performing EHIDA, concomitant heart disease). Continuous and categorical variables were expressed as medians with range and frequency with percentage respectively. All descriptive analysis and statistical calculations were performed with SPSS Statistics Version 24 (SPSS Inc., Chicago, IL., USA). This study was performed in accordance with the Declaration of Helsinki.

## Results

### EHIDA Results in All Patients

There was a total of 93 patients who underwent EHIDA during the study period, of which 32 (34.4%) were male and 61 (65.6%) were female. The median age at performing the scan was 45 days of life. We first analyzed the based on the diagnosis. Fifty-seven (61.3%) patients were confirmed to have BA and 36 (38.7%) had other causes of cholestasis including neonatal hepatitis and choledochal cysts. Among the 57 BA patients, 52 (91.2%) and 5 (8.8%) had positive and negative EHIDA results respectively. For the 5 EHIDA negative cases, two of them had an early EHIDA examination (< the median time of 45 days of life) while the other 3 had EHIDA after 45 days of life (on day 56, 57 and 109 respectively).

We then performed the analysis based on the EHIDA findings. EHIDA was considered positive in 59 patients, of which 56 (94.9%) subsequently proceeded to surgical exploration at a median age of 65 days of life, while the median age for all patients was 59 days of life. Fifty-two (92.9%) patients were eventually diagnosed with BA. For the 3 EHIDA positive patients who did not undergo surgical exploration, cholestasis resolved in two patients prior to surgery while the other one defaulted our subsequent follow up. Out of the 34 EHIDA negative patients, 9 (26.5%) of them required surgical exploration and 5 (14.7%) of them were subsequently confirmed to suffer from BA.

The sensitivity, specificity, NPV and PPV of EHIDA scan in BA diagnosis were therefore 91.2, 80.6, 85.3, and 88.1% respectively (Table 1).

**Table 1:** Correlation between EHIDA results and the diagnosis.

	**BA positive**	**BA negative**
EHIDA positive	52	7
EHIDA negative	5	29

*A positive EHIDA result means “BA cannot be excluded” due to the absence of Mebrofenin in the intestines in the 24-h delayed image; whereas a negative EHIDA result means either the presence of tracer activities in the intestines after 24 h or inconclusive findings, suggesting BA was unlikely*.

### Analysis of Factors That May Affect the Accuracy of EHIDA

#### Prematurity

Prematurity was defined as delivery before 37 weeks of gestation. In our study, 18 (19.4%) were premature babies while 75 (80.6%) were born at term. Among the premature patients, there were 1 false negative (5.6%) and 4 false positives results (22.2%). As for the term babies, there were 4 false negative (5.3%) and 3 false positive (4%) results (Table 2).

**Table 2:** Diagnostic accuracy of EHIDA in the context of different variables (Prematurity, age of life to conduct EHIDA, conjugated bilirubin level and concomitant heart disease).

	***N*** **(%)**	**True positive rate (%)**	**False positive rate (%)**	**True negative rate (%)**	**False negative rate (%)**
**Prematurity**					
Premature	18 (19.4%)	22.2	22.2	*50*	5.6
Term	75 (80.6%)	64	*4*	*26.7*	5.3
**Timing of EHIDA** (days of life)					
≤ 45	48 (51.6%)	64.6	2.1	29.2	4.2
> 45	45 (48.4%)	46.7	13.3	33.3	6.7
**Level of conjugated bilirubin (**μmol/L)					
≤ 66	48 (51.6%)	31.3	14.6	54.2	0
>66	45 (48.4%)	82.2	0	6.7	*11.1*
**Concomitant heart disease**					
Present	14 (15.1%)	*21.4*	14.3	*64.3*	0
Absent	79 (84.9%)	*62.2*	6.3	*25.3*	*6.3*

*N, Number of corresponding subjects*.

#### Age of Life to Conduct EHIDA

The age to conduct EHIDA of our subjects ranged from 10 to 110 days of life. We have classified them into examination before or after 45 days of life according to the median value for all ages of examination. For the 48 patients (51.6%) who had EHIDA before 45 days of life, there were 2 false negative (4.2%) and 1 false positive result (2.1%). For the 45 patients (48.4%) who had EHIDA after 45 days of life, there were 3 false negative (6.7%) and 6 false positive (13.3%) results respectively.

#### Conjugated Bilirubin Level

The conjugated bilirubin level prior to EHIDA scan of our subjects ranged from 10 to 238 μmol/L. We have divided our patients into 2 groups according to the median value of conjugated bilirubin level latest to performing EHIDA. For the 48 patients (51.6%) who had conjugated bilirubin level of <66 μmol/L, there were no false negative but 7 (14.6%) false positive results. For the remaining 45 patients (48.4%) who had conjugated bilirubin of more than 66 μmol/L, there were 5 (11.1%) false negative but no false positive results respectively.

#### Concomitant Heart Disease

There were 79 patients (84.9%) without concomitant heart disease. For the 14 patients (15.1%) who suffered from concomitant heart diseases, their cardiac diagnoses were valvular heart disease such as patent ductus arteriosus and atrial/ ventricular septal defects and cardiac arrhythmias. Among those with concomitant heart disease, there were 2 false positives (14.3%) but no false negative results. As for those without heart disease, there were 5 false positive (6.3%) and negative results.

## Discussion

EHIDA scan is a common investigation ordered for suspected BA. Many clinicians still depend on its result before the activation of BA management protocol. However, our data have revealed that this investigation is inaccurate for the diagnosis of BA, which reinforces the findings from previous studies (8). Among all parameters, the specificity is the lowest, suggesting the absence of radiotracer in the intestines should not be regarded as the pathognomonic of BA. More importantly, patients with EHIDA positive result should still be surgically explored for the definitive diagnosis (3, 9). On the other hand, false negative result can potentially delay the diagnosis of BA and the narrow treatment window by KPE is missed. Misinterpretation of EHIDA should also be considered. It has been reported that tracer activities in the urinary bladder and kidneys were mistaken as bowel activities in previous studies (6, 10).

In some centers, the availability of radionuclide scan is limited and takes time to be scheduled. KPE could have been delayed should BA be the underlying diagnosis and this is unfavorable as the prognosis is influenced by the timing of surgery. A better alternative is to proceed to surgical exploration with high clinical suspicion of BA after initial non-invasive and readily available investigations such as liver function test and abdominal ultrasound. Although there are additional surgical and anesthetic risks associated with surgical exploration in comparison to the use of EHIDA, the highest diagnostic accuracy has outweighed the disadvantages as BA is a lethal condition. With the advances of minimally invasive surgery in infants, surgical trauma is minimized. KPE can also be readily performed once the diagnosis is confirmed. If BA is excluded, intra-operative liver biopsy, which is often required to look for alternative diagnosis for the liver dysfunction, can be performed during the same operation. In our opinion, laparoscopic exploration can be considered a tool to achieve an earlier diagnosis of BA.

Despite a diminishing role of EHIDA scan in the diagnosis of BA, we attempted to identify the factors that may influence the accuracy of this investigation. Prematurity was evaluated because it may affect the hepatic uptake of radiotracer. Both the true and false positive rate were low in premature patients that suggested a higher diagnostic inaccuracy among premature patients. A postulation is that premature patients have underdeveloped liver function that reduces the hepatic uptake of the radiotracer during EHIDA study, leading to potential inaccuracy in evaluating the tracer activities in the bile ducts and intestines. On the other hand, as BA is a disease that evolve during peri-natal period, the biliary obstruction could have happened after the EHIDA scan is performed. However, there was an unequal distribution of patients across the two groups and further analysis with more evenly distributed sample is required. We also evaluated the impact of conjugated bilirubin level because of a postulation that Mebrofenin only allows biliary visualization on EHIDA images with bilirubin level up to 30 to 40 mg/dL, which suggested that a grossly elevated bilirubin level decreases the diagnostic accuracy of EHIDA (11). Milder jaundice seems to have a higher chance of false positive result. We next analyzed if the age of examination affects the accuracy of the scan. We found out that a delayed scan may result in higher false positive result. In advanced liver disease, the hepatic uptake an excretory function could be impaired that give rise to a false positive result. Last but not least, we evaluated if concomitant heart disease may affect liver function due to cardiac cirrhosis. Although there were differences in comparisons for this factor, as in the comparison for maturity, we could not draw a definitive conclusion due to the unequal distribution of samples.

As the passage of white stool is the first noticeable features of BA, stool color screening has been introduced in some regions as a non-invasive modality to screen for BA. It has been adopted as a screening programme in Taiwan and a high accuracy has been reported (12). This policy has also led to earlier diagnosis, treatment by Kasai operation and subsequently improved the 3-year jaundice-free survival in their population. Subsequently, the programme has been extended to other places that show similar efficacy. However, its applicability in regions with low prevalence of BA is limited due to a doubtful cost-effectiveness. Another issue concerns the compliance of using stool color cards and attending follow-ups for further management as it involves parents' incentive to use them. Nevertheless, we feel that for jaundiced-baby passing white color stool, direct proceed to surgical exploration without waiting for EHIDA scan should be considered.

The utilization of multiple non-invasive tests has been proposed to achieve an early diagnosis of BA (13). An example of this suggestion is the combination of B-US (high likelihood ratio+) and MRCP/hepatobiliary scintigraphy (low likelihood ratio-). The advantages of dual tests are the non-invasiveness in nature as well as improved diagnostic performances (14). However, there are also potential problems including the limited availability of MRCP in public institutions which may also lead to the delayed diagnosis of BA.

This study has several limitations. First, the study was only conducted in a single center and the analysis was performed in a retrospective manner. Second, the decision to refer a patient for EHIDA scan is subjective and there might be some patients who suffered from cholestasis but were not scanned and hence not evaluated in this study. The interpretations of the scan were done by a few radiologists and the interpretation of tracer activities in the intestines can be inconsistent throughout the study period.

## Conclusion

In conclusion, the accuracy of EHIDA scan in the diagnosis of BA is limited and maybe affected by prematurity, age at testing and the severity of cholestasis during examination. Future study with larger and more evenly distributed samples is required to identify factors that would improve its accuracy. For the best outcome of the patients, the decision of surgical exploration should not be delayed by EHIDA scan.

## Data Availability Statement

The raw data supporting the conclusions of this article will be made available by the authors, without undue reservation.

## Ethics Statement

Ethical review and approval was not required for the study on human participants in accordance with the local legislation and institutional requirements. Written informed consent from the participants' legal guardian/next of kin was not required to participate in this study in accordance with the national legislation and the institutional requirements.

## Author Contributions

WC manuscript writing, data acquisition, and data analysis. PC contributed to study design and critical revision. KW supervised the study. All authors contributed to the article and approved the submitted version.

## Conflict of Interest

The authors declare that the research was conducted in the absence of any commercial or financial relationships that could be construed as a potential conflict of interest. PC is a guest co-editor in this series KW is Associate Editor of Frontier in Pediatrics.

## Publisher's Note

All claims expressed in this article are solely those of the authors and do not necessarily represent those of their affiliated organizations, or those of the publisher, the editors and the reviewers. Any product that may be evaluated in this article, or claim that may be made by its manufacturer, is not guaranteed or endorsed by the publisher.
